# Association of Asthma and Allergic Rhinitis With Sleep-Disordered Breathing in Childhood

**DOI:** 10.3389/fped.2018.00250

**Published:** 2018-09-11

**Authors:** Evanthia Perikleous, Paschalis Steiropoulos, Evangelia Nena, Maria Iordanidou, Argyrios Tzouvelekis, Athanasios Chatzimichael, Emmanouil Paraskakis

**Affiliations:** ^1^Master Program in Sleep Medicine, Medical School, Democritus University of Thrace, Alexandroupolis, Greece; ^2^Department of Pneumonology, Medical School, Democritus University of Thrace, Alexandroupolis, Greece; ^3^Laboratory of Hygiene and Environmental Protection, Medical School, Democritus University of Thrace, Alexandroupolis, Greece; ^4^Department of Pediatrics, Medical School, Democritus University of Thrace, Alexandroupolis, Greece; ^5^Division of Immunology, Biomedical Sciences Research Center Alexander Fleming, Athens, Greece

**Keywords:** asthma, children, allergic rhinitis, sleep-related breathing disorder, pediatric sleep questionnaire

## Abstract

**Objective:** Asthma and allergic rhinitis (AR) are the most common chronic conditions in childhood and have previously been linked to sleep-related breathing disorder (SRBD). Aim of the study was to examine the association between SRBD risk and asthma control in children with asthma and with or without AR.

**Methods:** The assessment of FeNO and pulmonary function tests were performed in 140 children (65 with asthma, 57 with both asthma, and AR, 18 with only AR). Children with asthma completed the childhood Asthma Control Test (c-ACT), and the Sleep-Related Breathing Disorder scale, extracted from the Pediatric Sleep Questionnaire (PSQ). C-ACT scores ≤ 19 are indicative of poor asthma control whereas SRBD from PSQ scores ≥ 0.33 are suggestive of high risk for SRBD.

**Results:** Mean age ± SD was 7.8 ± 3.1 years. Mean PSQ ± SD and c-ACT ± SD scores were 0.17 ± 0.14 and 24.9 ± 3.2, respectively. High risk for SRBD was identified in 26 children. Children at high risk for SRBD had significantly decreased c-ACT score (*P* = 0.048), verified by a negative association between c-ACT and PSQ-SRBD scores (*r* = −0.356, *P* < 0.001). Additionally a difference in diagnosis distribution between children at high or low risk for SRBD was observed. More specifically, among children at high risk, 88.5% were diagnosed with both atopic conditions, while this percentage among children at low risk was 29.8%. Asthma was mainly diagnosed in the latter group (*P* < 0.001).

**Conclusions:** Poor asthma control is associated with SRBD. The presence of AR in children with asthma seems to increase the prevalence of SRBD in that particular population, requiring further investigation toward this direction.

## Introduction

Asthma is the most common chronic disease among children and has a significant financial impact in the Western world, especially in European countries (1), asserting that asthma control is of cardinal importance for public health. Management of symptoms and comorbidities are of fundamental importance for disease control (2). There is considerable evidence to support the interdependence of lower and upper airway; thus the umbrella term “united airway disease” is opportune. Epidemiological data indicate a correlation between allergic rhinitis (AR) and asthma (3, 4). Children with asthma and AR, exhibit poorer asthma control, reduced quality of life, increased risk for emergency visits, or hospitalizations, and higher health care burden (5, 6).

The link among asthma and sleep-related breathing disorder (SRBD) is bidirectional due to common risk contributors that, finally, induce airway inflammation (7, 8). SRBD in children with asthma may lead to difficult-to-control asthma. A recent study showed that SRBD is a robust risk factor for not-well-controlled asthma; through multivariate logistic regression analysis, the researchers have shown that the coexistence of SRBD and tonsillar hypertrophy were independent risk factors for not-well-controlled asthma after adjusting for other established factors to asthma control (9).

AR and SRBD in children are firmly related, and each medical condition can be detrimental to the other. Thusly, clinicians should be alert to the possibility of AR in patients with SRBD and vice versa. AR may influence sleep by several mechanisms. Nasal congestion due to the nasal mucosa allergic inflammatory procedure promotes increased airway resistance and may lead to oral breathing pattern, sleep fragmentation and excessive fatigue (10). Furthermore, inflammatory mediators of the allergic process could affect straightly the central nervous system by changing sleep circadian rhythm (10). It has been currently noticed, that the existence of AR in children with sleep disorders decreases Rapid Eye Movement (REM) sleep duration (11). In a recent study of children with moderate-to-severe persistent AR, even when submitted to treatment, demonstrated a higher prevalence of sleep disorders than the control group, principally night-time breathing disorders, daytime sleepiness, and parasomnias (12). In another, multicenter study, accomplished in various Latin-American centers, it was revealed that children with asthma and/or AR had a higher prevalence of sleep disorders in comparison to healthy controls (13).

Current asthma guidelines strongly emphasizes the importance of disease control assessment tools; yet, the monitoring of asthma control represents a major bottleneck for primary care physicians due to its multidimensional nature (14). Regarding children's, the childhood Asthma Control Test (c-ACT) is in alignment with asthma guidelines and represents one of the most extensively validated prognostic modality tools (15). FeNO is an important parameter of asthma management since many specialists have been using treatments applied in accordance to inflammatory markers, such as FeNO (16, 17). A recent meta-analysis suggests that using FeNO to guide treatment decisions may result in a lower rate of exacerbations, however it has trivial clinical advantage and the authors concluded that guideline-based asthma management and diagnosis constitute the optimal option (18).

Sleep disturbances occur frequently during childhood, affecting 15 to 30% of preschool children (19), and leading to a worldwide recognition as a crucial public health issue (20, 21). On top of that, the number of childhood sleep questionnaires has dramatically increased over the past few years (22). However, only a few have been validated by using standardized psychometric criteria, including the broadly used Pediatric Questionnaire for Sleep (PSQ) (23) and the SRBD-Scale, extracted from the PSQ (23).

The purpose of this preliminary study was to examine the association between SRBD risk and asthma control in children with asthma and with or without AR. As assessed by the following readout assays: (1) SRBD-Scale of PSQ, (2) levels of FeNO using the joint ATS/ERS guidelines (24).

## Methods

### Patients

The participants were consecutive individuals, aged between 4 and nearly 12 years, who suffered from asthma, AR or both disease entities. All of them had visited the Asthma Outpatient Unit of a tertiary hospital in Greece, as part of their routine follow-up, within 11 months, during the period between February 2014 and January 2015.

Asthma was usually suspected based on a typical history taken from every child, followed by pre/post bronchodilator spirometry test. To diagnose asthma in school-aged children (5 years and older) we used the ATS/ERS recommendations (25). For the younger, preschool children, the diagnosis was suspected based on the NHLBI's asthma research networks instructions, considering a positive modified asthma predictive index (API) if the child had experienced at least 4 exacerbations of wheezing in the past year, each episode lasting more than 24 h, and the following major or minor criteria: one of the major criteria; parental physician diagnosed asthma, physician diagnosed atopic dermatitis, evidence of sensitization to one or more aeroallergen, (i.e., positive skin tests or blood tests to allergens such as grasses), or two minor criteria; wheezing apart from colds, peripheral blood eosinophils ≥4%, evidence of food allergies (26, 27). The diagnosis was also confirmed by treatment responses. The diagnosis of AR was performed according ARIA guidelines (28). Findings of AR were consistent with one or more of the following symptoms nasal congestion, runny nose, itchy nose, and sneezing, red and watery eyes.

Children with chronic conditions, other than atopic diseases, essentially craniofacial abnormalities, neuromuscular disorders and/or genetic syndromes and children on any regular medication, other than long term control or short term asthma and AR medications, were excluded. Similarly, children who either they or their parents had been unable to communicate in Greek, and who have expressed a reluctance to participate were also excluded from the study.

The study protocol has been approved by the institutional board of ethics, and parental consent has been obtained for participation in the study, after being informed for the study goals.

### Procedures

Comprehensive medical history was taken from all participants. Informations were collected on exacerbation frequency, night-time symptoms, use of inhaled bronchodilators or corticosteroids, and history of any previous inpatient hospitalization due to asthma. A physical examination was also been performed.

Additionally, anthropometric parameters were determined. The BMI z-score, according to the age and sex of each subject, was calculated (29). The respiratory function was evaluated by performing spirometry as previously recommended and described (2). Furthermore, FeNO was assessed using a conventional chemiluminescence FeNO analyser (ANALYZER CLD 88sp, ECO MEDICS, Switzerland) (24). All the above mentioned procedures have been adhered to standard biosecurity and institutional safety procedures.

### Questionnaires

The 22-item SRBD subscale, extracted from the PSQ, was filled out by parents. We have used a Greek, non-validated version. The PSQ-SRBD questionnaire is assessing the presence of high risk for respiratory sleep disorders in children aged 2 to 18 years (30). The exported final score ranges from 0 to 1. Total scores ≥ 0.33 are considered positive and suggestive of high risk for pediatric SRBD (30).

A special version of ACT for children aged older than 4 and younger than 12 years, the c-ACT, complemented by parents and children with asthma (15). A list of c-ACT translations exists, but not all the listed questionnaires have undergone a full linguistic validation process. The Greek version of c-ACT is not validated. The c-ACT is divided into two separated parts; the first part is supplemented by the child and consists of four images. The gradation of the responses from the first part ranges from 0 to 3. The second part is answered by the escorting parent or guardian and consists of three other components ranging from 0 to 5. The total score of c-ACT is the sum of all responses, ranging from 0 which corresponds to the poorest asthma control, up to the value 27 which represents the optimal control of asthma (15). A value ≤19 indicates uncontrolled asthma.

### Statistical analysis

All data were checked for normality with the Kolmogorov-Smirnov test. For normally distributed variables, parametric statistics were used; conversely, nonparametric statistics were used when the distribution of data was not normal. All continuous variables are expressed as mean ± SD, at parametric statistics; and median and range (min, max) at nonparametric statistics. Correlations were investigated with Pearson or Spearman coefficient depending on the normality of the distribution. Significance was defined at the 5% level (*P* < 0.05). Analysis was performed using SPSS version 17 (IBM SPSS).

## Results

Out of a total consecutive 158 children who visited the Asthma Outpatient Unit of the University Pediatric Clinic during study, 140 met the inclusion criteria and, therefore, constituted the study sample. Among all participants, 65 had asthma alone, 57 had both asthma and AR, and 18 had AR alone. In particular, from the 18 excluded children; 3 were suffering from chronic conditions, other than atopic diseases, 5 had communication issues and 10 were excluded due to their parents' unwillingness to take part in the study.

Baseline characteristics of the studied population are listed in Table 1. The majority of subjects were boys (*n* = 89, 63.6%), and had the following characteristics: mean age ± SD 7.8 ± 3.1 years, mean PSQ result ± SD 0.17 ± 0.14, mean c-ACT score ± SD 24.9 ± 3.2, and a mean BMI z-score ± SD 0.97 ± 0.99. All children had normal lung function values.

**Table 1:** General characteristics of the enrolled children.

**Characteristics**	**Mean**	**Standard deviation**
Age (years)	7.8	3.1
PSQ-SRBD result	0.17	0.14
ACT score	24.9	3.2
BMI (Kg/m^2^)	19.1	3.3
BMI z-score	0.97	0.99
FVC (%pred)	93	12.3
FEV_1_ (%pred)	101.8	14.2
PEF (%pred)	86.8	15.6
FEF25-75 (%pred)	103.9	23.5
FeNO50 (ppb.10^−9^)	50.4	70
NO alveolar	3.1	1.7
NO bronchial	3598.1	4780.9

*BMI, Body Mass Index; c-ACT, childhood-Asthma Control Test; FEF25-75, Forced Expiratory Flow between 25 and 75% of vital capacity; FeNO50, Fractional Exhaled Nitric Oxide measured at a flow rate of 50 mL/s; FEV_1_, Forced Expiratory Volume in 1 second; FVC, Forced Vital Capacity; NO, Nitric Oxide; PEF, Peak Expiratory Flow; PSQ, Pediatric Sleep Questionnaire; %pred, % predicted value; ppb, parts-per-billion, 10^−9^*.

Twenty six children were identified as high risk for SRBD group (mean PSQ ± SD 0.4 ± 0.08). Comparison between these 26 children and children with normal PSQ rating did not reveal statistically important differences in terms of age (*P* = 0.858), FeNO value (*P* = 0,613), or pulmonary function tests (Table 2).

**Table 2:** Differences between children with high/low risk for SRBD.

**Characteristics**	**High risk (*n* = 26)**	**Low risk (*n* = 114)**	***P* value**
Age (mean ± SD)	7.8 ± 3.3	7.9 ± 3.1	0.858
BMI (mean ± SD)	18.1 ± 2.4	19.3 ± 3.5	0.105
FVC (mean ± SD)	90.3 ± 12.4	93.7 ± 12.2	0.251
FEV_1_ (mean ± SD)	99.9 ± 12.2	102.2 ± 14.6	0.487
PEF (mean ± SD)	86.6 ± 16.3	86.8 ± 15.5	0.961
FEF25-75 (mean ± SD)	105.9 ± 19.2	103.4 ± 24.6	0.655

*BMI, Body Mass Index; FEF25-75, Forced Expiratory Flow between 25 and 75% of vital capacity; FEV_1_, Forced Expiratory Volume in 1 second; FVC, Forced Vital Capacity; PEF, Peak Expiratory Flow*.

We performed a statistical analysis (*t*-test), comparing children with poor asthma control (c-ACT ≤ 19; *n* = 9) with children with adequate/good control (c-ACT >19; *n* = 113) in terms of PSQ-SRBD scores. The results were the following: In children with poor asthma control (c-ACT score = 16.5 ± 2.46): PSQ-SRBD 0.308 ± 0.183; in children with good asthma control (c-ACT score = 25.4 ± 2.21): PSQ-SRBD 0.158 ± 0.136 (*P* = 0.002). Of note, total scores ≥ 0.33 are considered positive and suggestive of high risk for pediatric SRBD. This analysis indicated that in poor asthma control PSQ-SRBD values are higher and tend to show positive SRBD A limitation in this analysis was the very small number, only 9, of children with c-ACT ≤19.

**Figure 1:**
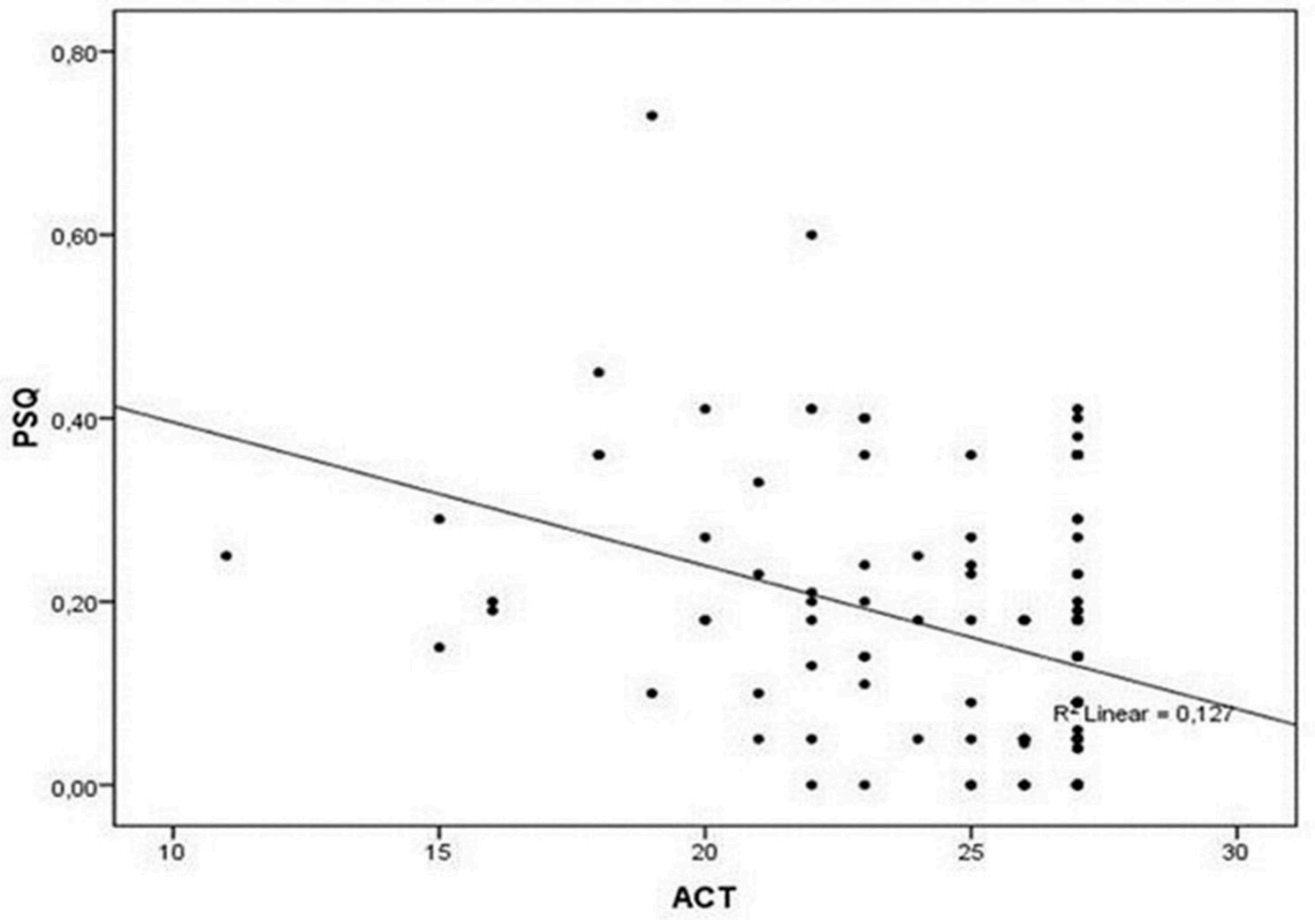
Correlation between PSQ and ACT scores. A negative linear correlation was found between the c-ACT and PSQ-SRBD scores (*r* = −0.356, *r*^2^ = 0.127, *P* < 0.001). ACT, Asthma Control Test; PSQ, Pediatric Sleep Questionnaire.

A statistically significant difference was noticed in mean values of c-ACT between the two subgroups (25.1 ± 3.1 vs. 23.7 ± 3.5, *P* = 0.048), reporting a correlation between asthma control and less likelihood of SRBD. The difference between them is small, with a *P*-value of 0.048, which, although is statistically significant, is very close to 0.05. We have the impression, however, that this difference is indicative of a trend toward poorer asthma control in children with SRBD, which could be more powerfully verified by studies including larger samples. At the same time, a negative linear correlation was found between the c-ACT and PSQ-SRBD scores (*r* = −0.356, *r*^2^ = 0.127, *P* < 0.001; Figure 1).

**Figure 2:**
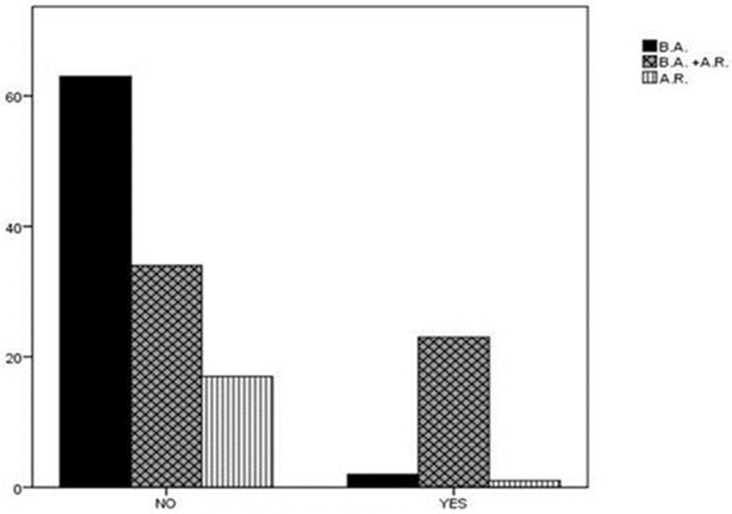
Diagnosis distribution in children with high or low risk for SRBD. In the subgroup of children with high risk for SRBD, 88.5% of children were diagnosed with asthma and AR. In contrast, the rate of coexistence of asthma and AR among children with low PSQ-SRBD score was 29.8%. AR, Allergic Rhinitis; BA, Bronchial Asthma; SRBD, Sleep Related Breathing Disorder.

Finally, a statistically significant difference was found in the diagnosis distribution of either asthma or AR or simultaneous presence of the two conditions between the two subgroups of children with high or low risk for SRBD. Specifically, in the subgroup of children with high risk for SRBD, the majority (*n* = 23, 88.5%) were simultaneously diagnosed with asthma and AR, only two were suffering from asthma alone, while only one from AR alone. In contrast, the rate of coexistence of both atopic diseases among children with low PSQ-SRBD score was 29.8% (*P* < 0.001; Figure 2). The percentage of children in high risk for SRBD (*n* = 26) in the whole group of participants (*n* = 140) was 18.5%.

## Discussion

In the present study we have demonstrated that poor asthma control is associated with high risk for SRBD in children. Additionally, among the majority of children with high risk for SRBD both asthma and AR were observed, compared to children with low risk, where asthma without AR was more prevalent. To our best knowledge, this is the first study in European pediatric population showing that inadequate asthma control and concomitant presence of AR are associated with increased risk for SRBD.

Few former studies in this emerging scientific field, have reported that children with asthma and AR exhibit poorer asthma control and higher risk of exacerbations, impaired life quality and increased healthcare cost compared to individuals without AR (5, 31, 32). Of note, nearly 80% of children with asthma exhibit concomitant AR, and ~40% with AR exhibit concomitant asthma (5, 6, 33). Previous studies have shown that appropriate quality and adequate quantity of sleep are crucial factors for many aspects of childhood health and development; however, more than 25% of children experience some type of sleep disorders (34). Accordingly, aggravation of the already impaired sleep quality is resulting in an increased night-time activity levels, leading to a vicious circle of further relapses of day-time sleepiness (34). Hence, a study shedding new light on the relationship between asthma control, AR and SRBD in children was sorely needed.

Our findings are in line with previous pediatric studies, showing a positive correlation between risk for Obstructive Sleep Apnea (OSA) and/or snoring and presence of asthma and/or wheezing (35–42). A random sample survey of 1234 children from Belgium, aged 6 to 14 years revealed a two-fold increased of OSA symptoms in children with wheezing (35). Similar findings have been reported in other countries (37, 38). Furthermore, in a group of African-American children with asthma, apnea-hypopnea index (AHI) was significantly higher in the subjects with inadequate asthma control (39). Additionally, a recent study enrolling Latin-American children from nine countries, with persistent asthma and/or AR as well as healthy controls, which filled the Children's Sleep Habits Questionnaire (CSHQ), concluded that especially uncontrolled asthma was leading to sleep impairment (13).

This study, though, was subjected to some limitations; therefore acknowledgment should be provided in order to allow interpretation of the described results. Firstly, as an observational, cross-sectional, pilot study with a small sample size, it cannot determine any causal relationship. Secondly, the risk of SRBD was assessed by a non-validated in Greek subjective instrument, such as SRBD subscale of the PSQ, and was not evaluated, thereafter, by a confirmatory objective tool, such as polysomnography. Thirdly, the evaluation of asthma control was based in c-ACT score, a non-validated in Greek composite control tool. However, although the idea of a score representing the overall asthma control seems attractive, it is universally accepted that asthma control represents a multidimensional process (14). The fact that both used questionnaires are non-validated in Greek, decreases the reliability of the study; the translation of both questionnaires into Greek and their reliability has to be determined by means of test-retest and internal consistency methods among a random sample of patients. Noteworthy, ACT is perhaps the most used subjective control tool in adults suffering with asthma and c-ACT represents one of the most extensively validated prognostic modality tools worldwide (15). C-ACT is beneficial in daily clinical practice based on its convenience to use, input from the child and caregiver, and alignment with asthma guidelines (15). SRBD subscale of the PSQ it can be valuable in clinical practice and research based on its ease of use and it can be useful for screening patients who require further medical evaluation, and for epidemiological reasons. Fourthly, we have only focused on asthma control and we did not examine the probable adverse effect of poor AR control on childhood SRBD. Nevertheless, this was the first European study underlining the importance of asthma control and, likewise, revealing the incremental role of AR in children with asthma, regarding the likelihood of SRBD. On the basis of the above-mentioned, more in depth knowledge about the need of multidisciplinary interventions integrating the co-management of asthma and AR, in order to diminish their impact on children's sleep is needed.

## Conclusions

In the present study we have shown that inadequate asthma control is associated with SRBD and the coexistence of AR in children with asthma seems to increase further the burden of SRBD. Monitoring asthma control is an integral part of asthma treatment, endorsing the importance of adequate control. In this term, periodic objective assessments of lung function may be deemed necessary to achieve the therapeutic goals set for individual child; although this was not found in this first pilot study; since no difference in lung function was shown between the two groups of high risk and low risk for SRBD. The coexistence of SRBD and asthma may have a cumulative effect, in terms of morbidity, so both disturbances must be recognized and treated promptly.

There is an urgent need for further, large scale research, in order to better understand the mechanisms between allergic disease and sleep. Undoubtedly, a systematic screening process including large epidemiological studies due to identify children at risk for SRBD is crucial in order to improve quality of medical care.

## Ethics statement

This study was carried out in accordance with the recommendations of Ethics Committee of Democritus University of Thrace with written informed consent from parent/guardians of all subjects. All parents gave written informed consent in accordance with the Declaration of Helsinki. The protocol was approved by the Ethics Committee of the Democritus University of Thrace.

## Author contributions

EvP collected the data, contributed in designing and drafting the manuscript. PS contributed in the initial conception and critical revision. EN analyzed the data, contributed in the design and interpretation. MI collected the data, drafted the initial manuscript. AT revised the manuscript and critical revision. AC drafted the initial manuscript and approved the final manuscript as submitted. EmP analyzed the data, supervised drafting of the initial manuscript and supervised revision of the manuscript. All authors provide their approval for the final version to be published.

### Conflict of interest statement

The authors declare that the research was conducted in the absence of any commercial or financial relationships that could be construed as a potential conflict of interest.
